# Automatic Visual Attention Detection for Mobile Eye Tracking Using Pre-Trained Computer Vision Models and Human Gaze

**DOI:** 10.3390/s21124143

**Published:** 2021-06-16

**Authors:** Michael Barz, Daniel Sonntag

**Affiliations:** 1 German Research Center for Artificial Intelligence (DFKI), Interactive Machine Learning Department, Stuhlsatzenhausweg 3, Saarland Informatics Campus D3_2, 66123 Saarbrücken, Germany; daniel.sonntag@dfki.de; 2 Applied Artificial Intelligence, Oldenburg University, Marie-Curie Str. 1, 26129 Oldenburg, Germany

**Keywords:** eye tracking, visual attention, eye tracking data analysis, area of interest, computer vision

## Abstract

Processing visual stimuli in a scene is essential for the human brain to make situation-aware decisions. These stimuli, which are prevalent subjects of diagnostic eye tracking studies, are commonly encoded as rectangular areas of interest (AOIs) per frame. Because it is a tedious manual annotation task, the automatic detection and annotation of visual attention to AOIs can accelerate and objectify eye tracking research, in particular for mobile eye tracking with egocentric video feeds. In this work, we implement two methods to automatically detect visual attention to AOIs using pre-trained deep learning models for image classification and object detection. Furthermore, we develop an evaluation framework based on the VISUS dataset and well-known performance metrics from the field of activity recognition. We systematically evaluate our methods within this framework, discuss potentials and limitations, and propose ways to improve the performance of future automatic visual attention detection methods.

## 1. Introduction

Eye tracking studies in many fields use Areas of Interest (AOIs) and visual attention to these AOIs as a common analytical helper tool. The resulting metrics are built to include events like AOI hits, dwells and transitions, which are based on raw gaze data or fixations with respect to a number of pre-defined AOIs. AOIs are tightly coupled to the hypotheses of a study because the corresponding metrics are used to argue for confirming or rejecting a hypothesis about visual stimuli. Hence, AOIs are very important, but incorrect placement of AOIs, and also inaccurate or imprecise mapping of gaze events to AOIs can heavily undermine the validity of a research study [1]. This adds the requirement of high robustness, accuracy, and precision for gaze estimation and gaze to AOI mapping methods. An AOI is usually defined as a spatial region with respect to the visual stimuli shown in a study, e.g., by defining a rectangular mask. For remote eye tracking with a static stimulus, it can be defined once and reused for every participant. The complexity increases if the stimulus is a video with dynamic AOIs, for example if they are linked to a dynamically moving object in the video. In this case, an AOI must be annotated for each video frame. This can be done frame-by-frame or, more efficiently, by defining bounding boxes for keyframes and interpolating intermediate frames [2]. Annotations can be reused if the video is the same for all participants: the participants’ individual gaze or fixation points can be mapped to these AOI regions automatically. In mobile eye tracking studies, each recording comes with an individual video. Hence, AOI definitions using frame-wise and keyframe-based annotation approaches cannot be reused which makes them inefficient. An alternative is the fixation-wise annotation: per fixation, an annotator has to decide whether an AOI is hit or not based on the visual stimulus around the fixation point [1]. A typical fixation lasts around 200–400 ms, which reduces the annotation effort compared to a frame-wise annotation. However, fixation-wise annotation does not remedy the need to annotate AOIs in every recording of every participant. A solution can be found in attaching fiducial markers to, e.g., a target stimulus in 2D [3] and 3D [4], an interactive area [5], or tangible objects [6]. In this research, we aim at circumventing the requirement to instrument the environment with obtrusive markers.

Previous methods tackled the automatic analysis of head-mounted eye tracking data in uninstrumented environments [7,8,9,10,11,12,13,14,15,16]. Drawbacks of these methods include, e.g., a missing support for real-time applications, and the restriction to a limited number of classes (≤12). Further, not all papers report quantitative evaluation results [7,8,14] or do not properly describe their evaluation metrics [9,10], or use inadequate metrics that ignore temporal aspects [12]. Some commercial tools offer automatic mapping of the gaze signal in world video coordinates to a reference frame that defines AOIs (For example, see the https://www.tobiipro.com/learn-and-support/learn/steps-in-an-eye-tracking-study/data/manual-and-assisted-mapping/, accessed on 15 June 2021). However, this is only possible for a limited number of reference frames.

In this work, we implement two methods for automating the detection of attention to task-related objects or AOIs in real-time. This can help in analyzing complex interaction behavior of humans: it bears the potential to facilitate novel real-time adaptive human-computer interaction [7,17], and to boost the efficiency in research based on eye tracking by automating the time-consuming and expensive data annotation process [12]. We contribute by (i) implementing two methods for detecting visual attention using eye tracking data and pre-trained deep learning models for image classification and object detection; and (ii) evaluating the performance of our methods using the VISUS dataset [2] and fine-grained activity recognition metrics in a systematic way [18]. The proposed evaluation framework and our results should serve as a reference for upcoming methods in automatic gaze to AOI mapping. Further, we discuss the performance of our methods and how interactive transfer learning can be used to break the limitations of pre-trained models.

## 2. Related Work

Our work aims at accelerating and objectifying research on visual attention with mobile eye tracking using technologies from the field of computer vision. In human perception, “selective visual attention is the allocation of limited attentional resources to certain information in the visual field, while ignoring other information” [1] (p. 26). It can be guided by salient bottom-up factors and task-related top-down factors in a scene [19]. When humans perform a task, the number of fixations to irrelevant but salient objects drop, while the fixations to task-relevant objects, i.e., top-down factors, increase [1,20,21,22,23]. In the following, we summarize related work that used human gaze for intelligent human-computer interaction, and we describe related approaches that addressed the problem of automatic or semi-automatic gaze-to-AOI mapping in non-instrumented environments. Further, we provide a brief overview on the state-of-the-art in computer vision in this regard.

### 2.1. Eye Gaze in Human-Computer Interaction

Human gaze, which can be seen as a proxy for human visual attention, can be beneficial when applied in intelligent human-machine interaction [24,25,26]. It can be used as an active or passive input modality [27]. For instance, a user can influence a system via explicit eye movements (active) and a system can implicitly derive information about the user, its state, and intentions by observing the eye movement behavior (passive). In this paper, we focus on eye gaze as an implicit source of context information. Related works in this field investigated and applied eye gaze in the context of conversational interfaces, information retrieval systems, and situation-aware human-machine interaction, in general.

Ishii et al. [28] proposed a system for estimating the user’s conversational engagement using eye tracking data from a Wizard-of-Oz study. In a subsequent work, they modelled turn-taking behavior in human-human dialogues based on eye gaze features [29]. A similar approach was presented by Jokinen et al. [30]. Prasov and Chai [31] developed a system that combines speech and eye gaze to enhance reference resolution in conversational interfaces. Xu et al. [32] investigated the role of mutual gaze in a human-robot collaboration setting and found that maintaining eye contact leads to improved multimodal interaction behavior of users, i.e., more synchronized and coordinated. Baur et al. [33] implemented NovA, a system for analyzing and interpreting social signals in multi-modal interactions with a conversational agent, which integrates eye tracking technology. Thomason et al. [34] developed a gaze-based dialog system that enables grounding of word meanings in multi-modal robot perception.

In the domain of information retrieval, Buscher et al. [35] investigated the relation between reading behavior and document relevance. The authors introduced the concept of attentive documents that keep track of the perceived relevance based on eye movements. Other works investigated the utility of eye tracking in multimedia retrieval settings. Several algorithms were proposed for estimating the search target of an ongoing visual search on a screen [36,37,38,39]. Barz et al. [40] introduced an algorithm for estimating the target segment of a visual search in more natural settings.

Eye tracking was also used to facilitate situation-aware human-machine interaction in general. Bulling et al. [41] presented an approach for inferring high level contextual cues from eye movements to facilitate behavioral monitoring and life-logging. Similarly, Steil and Bulling [42] used topic modeling to detect everyday activities from eye movements in an unsupervised fashion. In a later work, the authors presented an approach for visual attention forecasting in mobile interaction settings which takes the visual scene and device usage data as additional inputs [43]. Also, other works combine visual features of a scene with gaze information for recognizing recent actions [44,45,46,47]. In the context of human-robot interaction, Ramirez-Amaro et al. [48] showed that human behavior inference benefits from incorporating mobile eye tracking data with third person videos. Recently, Kurzhals et al. [49] described an interactive approach for annotating and interpreting egocentric eye tracking data for activity and behavior analysis. They implement an iterative time sequence search based on eye movements and visual features. Steichen et al. [50] investigates the effectiveness of eye tracking for predicting user characteristics like cognitive abilities and the utility of such a model in adaptive information visualization. A comparison of uni-modal and multi-modal methods for user modeling in the context of real-time adaptive data visualization can be found in [51]. These works aim at segmenting eye tracking recordings into phases of different activities. Our goal is to identify phases of attention to objects in a scene that can serve as AOI.

### 2.2. Gaze-to-AOI Mapping

A few works address the problem of mapping human gaze to objects or areas of interest in non-instrumented environments. Pontillo et al. [10] presented SemantiCode, an interactive tool for post-hoc fixation-based annotation of egocentric eye tracking videos. It supports semi-automatic labelling using a distance function over color histograms of manually annotated fixations. Brône et al. [14] proposed to use object recognition with mobile eye tracking to enhance the analysis of customer journeys. In follow-up works, they compared different feature extraction methods [52] and evaluated their approach in a museum setting [15]. Evans et al. [53] reviewed methods for mobile eye tracking in outdoor scenes ranging from pupil detection and calibration to data analysis. They presented an early overview of methods for automating the process of analyzing mobile eye tracking data. Fong et al. [54] presented a semi-automatic data annotation approach using on the human-in-the-loop principle. The annotator can label individual frames based on the visual appearance and the gaze position. As the annotation process advances, the system learns the appearance of AOIs based on examples and to automatically classify clearly similar cases. Panetta et al. [12] presented an annotation method based on bag-of-visual-words as features and a support vector classification model (SVC) that is trained before the analysis takes place. In a follow-up work, the authors present a system that automatically segments objects of interest using two state-of-the-art neural segmentation models [55]. They use pre-trained models to showcase and evaluate new data visualization methods, but they did not assess the performance of their automatic annotation approach. Kurzhals et al. [8] used bag-of-SIFT features and color histograms with unsupervised clustering to sort fixation-based image patches by their appearance. They offer an interactive visualization for manual corrections. Venuprasad et al. [13] use clustering with gaze and object locations from an object detection model to detect visual attention to an object or a face. Sümer et al. [56] investigated the problem of automatic attention detection in a teaching scenario. They extract image patches for all student faces in the egocentric video feed and cluster them using activations from a ResNet-50 [57] model trained on VGGFace2 data [58]. They assign student IDs to each cluster which allows them to map the teacher’s gaze to individual students. Callemein et al. [59] presented a system for detecting when the participant’s gaze focuses the head or hands of another person without the possibility to differentiate between interlocutors. Other works also focused on real-time applications. For example, Toyama et al. [11] implemented the Museum Guide that uses SIFT (scale-invariant feature transform) features [60] with the nearest neighbor algorithm and a threshold-based event detection to recognize user attention to one of 12 exhibits. They extended their approach to detect read texts and fixated faces with the goal to build artificial episodic memories to support dementia patients [61]. Barz and Sonntag [7] presented a similar approach using a GoogLeNet model [62] pre-trained with ImageNet [63] data. Wolf et al. [16] implemented the computational Gaze-Object Mapping algorithm that maps fixations to object-based AOIs using the Mask R-CNN object detection model [64]. They conducted a controlled lab study to record data in a healthcare setting with two AOIs, a *bottle* and five *syringes*. An evaluation has shown that, using 72 training images with 264 annotated object masks, their system can closely approximate the AOI-based metrics in comparison to manual fixation-wise annotations as a baseline. Batliner et al. [65] presented a similar system for simplifying usability research with mobile eye trackers for medical screen-based devices. Machado et al. [9] matched fixations with bounding boxes of another object detection algorithm to detect user attention. They used a sliding-window approach with a MobileNet model [66], pre-trained on ImageNet data, to detect objects in an image.

### 2.3. Computer Vision

Computer vision is the algorithmic equivalent to human visual perception and subsumes image classification and object detection methods. Image classification refers to the assignment of a single label to an image, object detection refers to the localization and classification of multiple objects in a single image [63]. Recent methods experienced a performance boost with the advance of deep learning technology and the availability of large datasets for model training. Popular examples are the ImageNet dataset for image classification [63] and the MS COCO dataset for object detection [67]. A recent overview of object detection with deep learning can be found in [68]. We apply residual network models introduced in [69], pre-trained on ImageNet, and the Mask R-CNN model for object detection [64], pre-trained on MS COCO. Related works also include methods for egocentric activity recognition without gaze data. For example, Ma et al. [70] use hand segmentations, object localizations and the optical flow from first-person videos to infer ongoing activities. Another example is EgoNet by Bertasius et al. [71] which determines the action-object in egocentric videos.

## 3. Method

**Figure 1:**
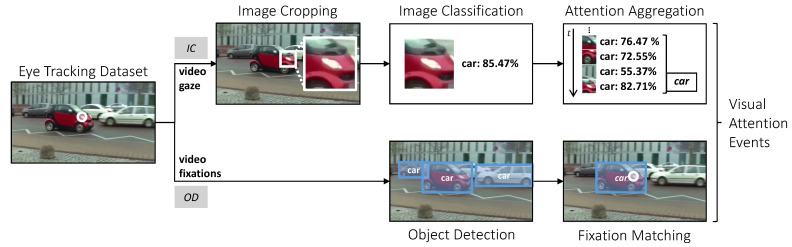
Processing workflow of the two proposed methods for automatic attention detection: *IC* is based of image classification and gaze samples, *OD* uses object detection and fixation events. Both methods support visual attention detection in real-time.

We implement two methods for an automatic detection of visual attention to a visual stimulus in a scene. Both take the video feed and the corresponding gaze or fixation signal as input and predict, if the participant paid attention to an AOI for each frame (see Figure 1). The first method, *IC*, aggregates classifications of image patches, cropped around the gaze signal, using a pre-trained image classification model similar to the gaze-guided object classification system by [7]. The second method, *OD*, matches fixation events with the result of a pre-trained object detection model similar to Wolf et al. [16] and Machado et al. [9]. In this work, we concentrate on pre-trained computer vision models, similar to Barz and Sonntag [7] and Machado et al. [9], to explore when models without a training overhead can be applied effectively and when they reach their limits. We leave fine-tuning of the models as a task for future-work, because it is outside the scope of this paper. Both methods are implemented in Python using the *multisensor-lpipeline* (https://github.com/DFKI-Interactive-Machine-Learning/multisensor-pipeline, accessed on 15 June 2021) package for flexible streaming and processing of signals from one or multiple sources. It allows to easily set up real-time applications using source modules for connecting sensor input, processor modules for manipulating or aggregating incoming data streams and events, and sink modules for, e.g., storing and visualizing the output. In the following, we describe the implementation of both methods and their adjustable parameters.

### 3.1. Detect Attention Using Gaze-Guided Image Classification (IC)

Our method based on image classification includes four subsequent steps. First we re-sample the gaze signal to 5 Hz and crop an image patch of 200×200 pixels from the egocentric video feed (1920×1080) per remaining sample. We use this crop size, because it turned out to perform well in real-time applications (see [7,72]) and the size fits well to the AOIs in the VISUS dataset (manual inspection). Second, each patch is classified using a pre-trained version of the ResNet image classification model [69] which is trained on the ImageNet dataset with 1001 object classes [63]. The prediction result includes the top-5 class candidates and their probability. In a third step, we aggregate same or similar class labels by accumulating their probabilities. We merge similar object classes based on a manually defined lookup table. For example, if the top-5 output includes the ImageNet classes passenger car, streetcar and limousine, we replace the probability of passenger car by the sum of all three probabilities and remove the remaining class labels from the output. In the last step, we implement a working memory- and threshold-based attention detection algorithm similar to Barz and Sonntag [7] and Toyama et al. [11] using the top-1 predictions of the previous step as continuous input:

An update routine is called for each incoming prediction, i.e., a tuple including a unique class label and the corresponding probability output of the model: (c,p(c)). If p(c) exceeds the minimum probability $T_{p}$, we increase the duration counter $C_{dur}$ at the index *c* by the amount of milliseconds that passed since the last run of the update loop (circa 200 ms). For all other classes with a non-zero duration count, we increase the noise counter $C_{noise}$ by the same amount of time. If the aggregated duration $C_{dur}\left[c\right]$ exceeds the duration threshold $T_{dur}$, we send an *attention started event* including *c*, p(c) and the timestamp of the latest prediction. In addition, we store *c* as the currently attended class $c_{active}$ and reset both counters for it: we set $C_{dur}\left[c\right]$ and $C_{noise}\left[c\right]$ to zero. If $c_{active}$ is not empty and not equal to *c*, we consider the prior attention event to be over and send an *attention ended event*. We send an *attention confirmed event* is *c* is equal to $c_{active}$. Finally, we check for all remaining classes whether the aggregated noise duration in $C_{noise}$ exceeds the noise threshold $T_{noise}$. In this case, we reset both counters for this class and, if this class is equal to $c_{active}$, we send an *attention ended event*. We subtract $T_{dur}$ from the event timestamp to better match the actual start and end times of the attention events. We offer a parameter for setting the image classification *model*: we include the ResNet-50 and ResNet-152 models via Tensorflow Hub. The pre-trained models can be found at https://tfhub.dev/google/imagenet/resnet_v2_50/classification/4 and https://tfhub.dev/google/imagenet/resnet_v2_152/classification/4, respectively (each accessed on 2 May 2021). Any other model from this platform, that was trained using ImageNet, can be used as well by providing a corresponding link. The default setting is $T_{dur}=T_{noise}=300$ ms, $T_{p}=40\%$, and the *model* is set to ResNet-152. We refer to this setting as IC-152-300-40 (in general: IC-*model*-$T_{dur/noise}$-$T_{p}$).

### 3.2. Detect Attention Using Object Detection (OD)

Our second method is based on an object detection model which can detect multiple object instances in an image from a set of candidate classes. To detect visual attention, we match the position of fixation events from the eye tracker with detected object regions. For each fixation, we extract an image frame from the video feed that is closest to the start of the fixation event. The object detection takes longer per image than the image classification algorithm. However, this method can still be applied in real-time, because it is applied once per fixation. During a fixation the eye is relatively still and, hence, should point to the same location in the world space. But, fixation detection is not perfect, e.g., in presence of smooth pursuit movements, which makes this method dependent on the quality of the applied fixation detection algorithm. Next, we detect all object instances in the current image frame: we use a Mask R-CNN model [57] that is pre-trained on the MS COCO dataset [67] with the Detectron2 framework [73]. The model weights can be downloaded from https://dl.fbaipublicfiles.com/detectron2/COCO-InstanceSegmentation/mask_rcnn_R_101_FPN_3x/138205316/model_final_a3ec72.pkl (accessed on 2 May 2021). For each instance, it provides a class label with a probability value, as well as a rectangular bounding box and a pixel-wise segmentation mask depicting the object area. Finally, we check whether the fixation position lies within the object area, either using the bounding boxes (*bbox*), similar to Machado et al. [9], or the more fine-grained segmentation masks (*mask*), similar to Wolf et al. [16], as reference. This can be configured via the *object mask* parameter that defaults to *bbox*. If a hit is detected, we send an *attention started event* using the start time of the fixation and an *attention ended event* using its end time. If two object areas are hit, we choose the one with higher probability. We refer to the two possible settings as OD-bbox (default) and OD-mask.

## 4. Evaluation

We evaluate the performance of the two methods described above in terms of their ability to detect time intervals in which a participant fixates a certain AOI. Our evaluation procedure utilizes the VISUS dataset [2] including eye tracking data from 25 participants for 11 scenarios, and manual AOI annotations which we use for ground truth extraction. To measure the performance, we use a set of frame- and event-based metrics by Ward et al. [18] from the field of activity recognition which allow a more fine-grained analysis. We report the metrics per scenario and for each of the 34 AOIs to identify effective applications and limitations.

### 4.1. Dataset

We use the VISUS dataset for our evaluation [2] which can be downloaded from https://www.visus.uni-stuttgart.de/publikationen/benchmark-eyetracking (accessed on 12 April 2021). It contains eye tracking data from 25 participants for 11 video stimuli, totaling to 275 sessions. The gaze data was recorded using a Tobii T60 XL remote eye tracker at 60 Hz. The authors did not report the spatial accuracy and precision as measured during their recordings. The video stimuli have a resolution of 1920×1080 pixels at 25 frames per second and have an average length of 75.55 s (SD=59). Each video is manually annotated with axis aligned rectangular bounding boxes from two annotators for 1 to 6 AOIs per video (see Table 1). Bounding boxes were set at key frames and interpolated for intermediate frames. The main purpose of the dataset is to serve as a benchmark for visualization and analysis techniques in the field of eye tracking. We use the dataset as a benchmark dataset for automatic detection of visual attention to dynamic AOIs. We treat the fixation events reported in the dataset that hit the manually defined bounding boxes as ground truth attention events to the respective AOIs. If two AOIs in a single frame are hit, we select the AOI that yields the longer event. While the VISUS dataset is acquired with a remote tracking device, we use it to approximate mobile eye tracking recordings: we do not leverage that the videos are the same for each participant. In the following, we describe the ground truth extraction, the scenarios (video stimuli) and AOIs, and we describe the related challenges for gaze to AOI mapping.

#### 4.1.1. Scenarios & Challenges

The dataset includes 11 scenarios each with a different kind and number of AOIs. They pose multiple challenges to attention detection methods. In the simplest case, a method has to map gaze to AOIs that represent distinct concepts (challenge I). This applies to, e.g., 01-turning car in which a single AOI, a “red car”, is shown, and to 07-kite with two distinct AOIs: a “person” flies a “kite”. The difficulty increases, if two AOIs in a scenario refer to the same concept (challenge II). For instance, the scenario 01-car pursuit shows a “red car” driving through a turning area, with a “white car” on the opposing lane and multiple parking cars in the background. The challenge is not only to detect that a car is fixated, but to differentiate between the two prominent cars (AOIs) and the background cars which are multiple instances of the same concept. Similarly, the scenarios 03, 08, 09, and 11 require the ability to differentiate between multiple instances of the concept person, for instance, a “hooded” person, a person wearing a “red shirt and hat”, and several distractor “persons” in scenario 11-person search. The problem complicates, if two AOIs not only share a concept, but also their appearance (challenge III). An example can be found in scenario 04-thimblerig which includes three cups with identical appearance. Distinguishing them requires object tracking for multiple instances and, hence, an initial assignment of each instance to an AOI by hand. The scenario 05-memory is not covered by the aforementioned cases. It shows a memory game: in the beginning, all 16 “cards” look the same, while, until the end of the game, we see 8 pairs of cards with different visual appearance per pair. Yet, all “cards” count toward the same AOI. The challenge is, if the appearance of an AOI changes over time (challenge IV).

#### 4.1.2. Mapping Class Labels to AOIs

Our methods aim at solving the aforementioned challenges using pre-trained computer vision models. For this, AOIs need to be mapped to class labels of ImageNet for the *IC* method and of MS COCO for the *OD* method. We assume that the performance per scenario depends on the type of AOIs and whether they are represented in the training data of the model. If there is no matching class label for an AOI, none of the methods can detect respective attention events. If a class label matches multiple AOIs of a scenario, i.e., if they share a concept, we can only assign the label to one of them. This probably leads to an increase in false positives. The performance might also suffer from inadequate matches. For this experiment, we use a separate mapping from class labels to AOIs for each method and scenario, as shown in Table 1. For *IC* methods, we identified ImageNet labels for 19 AOIs including adequate matches like *passenger car* for the AOI “red car”, but also weak matches like *sweatshirt* as a proxy for the AOI “person”. Similarly, we found MS COCO labels for 17 AOIs for the *OD* methods. For instance, *car* is an adequate match for the AOI “red car”, while *dining table* is a weak match for the “stack covered” in 06-UNO (the stack is located on a table).

### 4.2. Metrics

**Figure 2:**
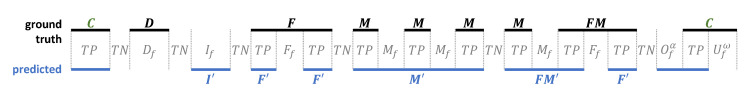
Example of segmented ground truth events and predicted events with annotations for event error and frame error classes. The vertical bars depict the segment boundaries. The frame error classes are given per segment.

To quantify the performance of our methods, we need evaluation metrics that depict how well our detected attention events match the ground truth events. We reviewed the metrics proposed in closely related works, but none of them was fully satisfactory: Panetta et al. [12] compared their system to manual ground truth annotations by calculating the distance between two histograms that aggregate the duration of fixations from predicted or ground truth AOI regions, respectively. However, their metric does not punish if detected AOI fixations are shifted in time or if they occur in the wrong order, which puts the validity of their metric into question. For instance, the histogram would be equal, if the predicted events were reported reversely. Machado et al. [9] reported accuracy and precision, but it is unclear whether they compute the metrics frame-wise or event-based. Toyama et al. [11] reported event-based precision and recall for each method: precision reflects how many of the detected attention events were classified correctly, recall indicates the proportion of detected attention events to all attention events. Similarly, De Beugher et al. [15] reported precision and recall, but at the frame-level. Wolf et al. [16] and Batliner et al. [65] reported the recall (true positive rate) and the specificity (true negative rate) at the frame-level, including one frame per fixation for the analysis. The specificity reflects the ratio of frames that are correctly classified as not showing human attention to an AOI (negative class) in relation to all frames with a negative class label. Sümer et al. [56] compared the absolute number of predictions for each class, i.e., four individual students, to the ground truth count. In addition, they use a confusion matrix to show the performance of their face recognition system that is used to assign fixations to students’ faces. Callemein et al. [59] used measures for inter-rater agreement like Cohen’s κ to show the performance of their gaze-to-face and gaze-to-hand mapping. Venuprasad et al. [13] reported precision, recall, and accuracy for frames, and event metrics based on detection events: first looks, extra looks (i.e., revisits), false positive and false negative events are counted. Other works reported qualitative results only or did not evaluate their method. In this work, we report fine-grained frame- and event metrics per AOI from the field of activity recognition [18]. They were shown to be effective for evaluating event detection methods in the field of mobile eye tracking [18,74]. The metrics are based on a segmentation of the ground truth and prediction signal at the frame level per AOI (see Figure 2). A segment ends, if the ground truth or the prediction changes, i.e., both signals are constant within a segment. Each segment can now be rated as one of true positive, true negative, false positive, or false negative. The event and frame metrics are derived from these segments. Prior to feature computation, we remove events with a duration smaller than the frame time and merge adjacent events.

#### 4.2.1. Event Metrics

Ward et al. [18] define a set of error classes for events which are meant to characterize the performance of a single-class event detection method. For multi-class problems, each class is handled separately. Error classes include the insertion ($I^{\prime }$) and deletion (*D*) error which are commonly used in event detection. An insertion error depicts that a detected event is not present in the ground truth (false positive), and a deletion error indicates a failure in detecting a ground truth event (false negative). Additional error classes include fragmentation and merge errors: a ground truth event is fragmented (*F*), if multiple fragmenting events ($F^{\prime }$) are detected in the output. Similarly, multiple ground truth events of the same class can be merged (*M*) by a single merging event ($M^{\prime }$) in the output. Both errors can appear together, e.g., if a ground truth event is fragmented by three event detections of which the third is merging an additional ground truth event. In this case, the first ground truth event is marked as fragmented and merged (FM), and the third event detection is marked as fragmenting and merging ($FM^{\prime }$). The apostrophe indicates whether an error class is assigned to a ground truth event or a predicted event in the output. If none of the error classes can be assigned, a detected event is counted as correct (*C*), i.e., as a true positive. According to Ward et al. [18], we visualize the metrics by means of an event analysis diagram (EAD). It shows the number and ratio of error classes in relation to the number of reference events, i.e, to the number of ground truth events |E|=D+F+FM+M+C, the number of predicted events (or returns) $\left|R\right|=M^{\prime }+FM^{\prime }+F^{\prime }+I^{\prime }+C$, or both in case of correct predictions *C*. Also, we can compute event-based precision and recall as a ratio between |R| or |E| and the error class counts. We compute a conservative precision as $Pr=\frac{C}{\left|R\right|}$ and recall as $Re=\frac{C}{\left|E\right|}$. Counting F,FM,M and $M^{\prime },FM^{\prime },F^{\prime }$ as correct, similar to Toyama et al. [11], we calculate a more progressive precision as $Pr^{*}=\frac{\left|R\right|-I^{\prime }}{\left|R\right|}$ and recall as $Re^{*}=\frac{|E|-D}{\left|E\right|}$.

#### 4.2.2. Frame Metrics

For extracting the frame metrics, Ward et al. [18] project error classes to frames per segment. Similar to event-based error classes, a frame can be rated as insertion ($I_{f}$), deletion ($D_{f}$), merge ($M_{f}$), or fragmentation ($F_{f}$). Merge errors are assigned to false positive frames from merging events and fragmentation errors are assigned to false negative frames between fragmenting events. Further, if a neighboring segment is classified as true positive, frames of a false positive segment are marked as overfill ($O_{f}$) and frames of a false negative segment are marked as underfill ($U_{f}$). In other words, an overfill occurs, if a detected event starts early or ends late, and an underfill occurs, if a detected event starts late or ends early. A superscript indicates whether an underfill or overfill occurs at the start (α) or end (ω) of an event. Frames of true positive (TP) and true negative (TN) segments are classified likewise. Ward et al. [18] define the frame metrics as ratios of the error class counts and the total positive frames *P* or negative frames *N* in the ground truth, with $P=D_{f}+F_{f}+U_{f}^{\alpha }+U_{f}^{\omega }+TP$ and $N=I_{f}+M_{f}+O_{f}^{\alpha }+O_{f}^{\omega }+TN$. The resulting ratios (lowercase equivalents to error classes) can be used to express the false positive rate as $fpr=ir+mr+o^{\alpha }+o^{\omega }$, and one minus the true positive rate as $\left(1-tpr\right)=dr+fr+u^{\alpha }+u^{\omega }$. We use a set of two stacked bar charts to visualize the frame metrics (compared to pie charts in [18]).

### 4.3. Experiment Conditions & Procedure

We compare two methods for visual attention detection: *IC* based on gaze-guided image classification and a threshold-based event detection, and *OD* based on object detection and fixation mapping. We generate predictions for the VISUS dataset using each method and analyze their results. We start with default parameters to identify AOIs which are not supported. We define cases with a recall of zero to be failing: this corresponds to a deletion rate of dr=100% (frame metrics), or if all ground truth events are marked as deletions *D*. By design, we expect AOIs without a matching class label to fail (see dashes in Table 1). For the remaining AOIs, we investigate the impact of different methods and parameters on the performance metrics. We compare two *IC* methods using the classification *models*, ResNet-50 and ResNet-152, and two *OD* methods using the *object mask* options bbox and mask. The other parameters are set to their defaults, which results in the following set of parameterized methods: IC-152-300-40, IC-50-300-40, OD-bbox, and OD-mask. For *IC*, we additionally test different values for $T_{dur}$, $T_{noise}$, and $T_{p}$ using the ResNet-152 *model*, with $T_{dur}=T_{noise}\in \left\{100,300,500,700\right\}$ ms and $T_{p}\in \left\{20\%,40\%,60\%\right\}$. Changing these parameters might have an effect on the performance of the *IC* method. Per method, we compute the frame and event metrics for each AOI and per participant. We sum the metrics over participants, if we report the performance per AOI, and over participants and AOIs, if we report the overall performance of a method. Summing the metrics corresponds to concatenating the recordings of all participants per AOI, because the metrics are based on absolute counts. Ratios are computed afterwards using the number of positive and negative ground truth frames or events which we add up as well.

### 4.4. Results

Using default parameters, we observe a recall of zero for all AOIs without a matching class label for a method, but also for other AOIs: for the *IC* method, this includes “left face”, “cup1”, “cards”, “stack covered”, “case”, “player white”, “red bag”, “brown bag”, and “hooded”. For the *OD* method, this includes “cup1”, “cup2”, “cards”, “stack covered”, “case”, “red bag”, and “persons” (for 10-bag search only). We count one additional AOI for *OD* (“ball”) and three AOIs for *IC* (“suspects”, “ball”, and “persons” in 10-bag search) as failing, because they yield a recall close to zero (dr≥90%). The remaining six AOIs for *IC* and nine AOIs for *OD* are analyzed in detail (AOIs are listed in Section 4.4.2). The AOIs for *IC* include |E| = 2438 ground truth events that correspond to *P* = 71,911 positive frames and *N* = 111,467 negative frames. The AOIs for *OD* include |E| = 4328 events with *P* = 154,783 positive and *N* = 270,750 negative frames.

#### 4.4.1. Overall Performance

**Figure 3:**
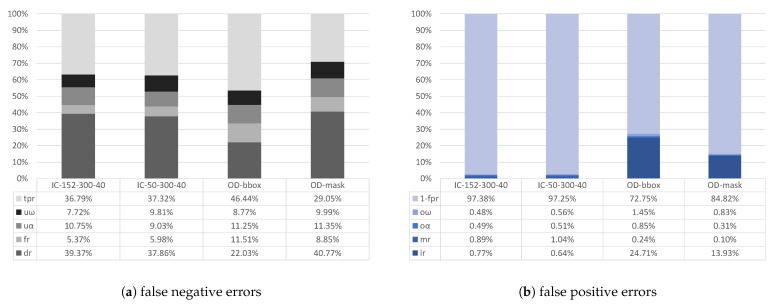
Frame metrics with respect to positive (**a**) and negative (**b**) ground truth frames across all AOIs.

We compare the metrics of two *IC* and two *OD* methods that vary in terms of the *model* or *object mask* setting (see Section 4.3). The frame metrics for remaining AOIs are summarized in Figure 3. It shows the ratios of false negative errors with respect to *P* in Figure 3a, and of false positive errors with respect to *N* in Figure 3b. Concerning the false negative errors, deletion is the most prominent class across all methods: they account for 22.03% (OD-bbox) and 40.77% (OD-mask) of the errors for *OD*, and dr is 39.37% for ResNet-152 and 37.86% for ResNet-50 for the *IC* methods. On average, the tpr does not differ between *OD* (37.75%) and *IC* (37.05%). However, OD-bbox yields the best tpr with 46.44%, which is 9.39% better than the average of both *IC* methods and 17.39% better than OD-mask. The remaining error classes account for 30.86% for *OD* and 24.33% for *IC*: on average, *IC* faces 6.53% less false negatives through fragmenting events and underfills than *OD*. Concerning the false positive errors (Figure 3b), insertions are most prevalent for *OD* with ir=24.71% for OD-bbox and ir=13.93% for OD-mask. For *IC*, we observe less insertions, averaging to 0.71%. Errors from merging event detections and overfills account for 1.98% (*IC*) and 1.89% (*OD*). Hence, the fpr adds up to 2.69% for *IC* and to 21.21% for *OD*, which means that the *OD* methods cause 18.53% more false positive errors at the frame level, on average.

**Figure 4:**
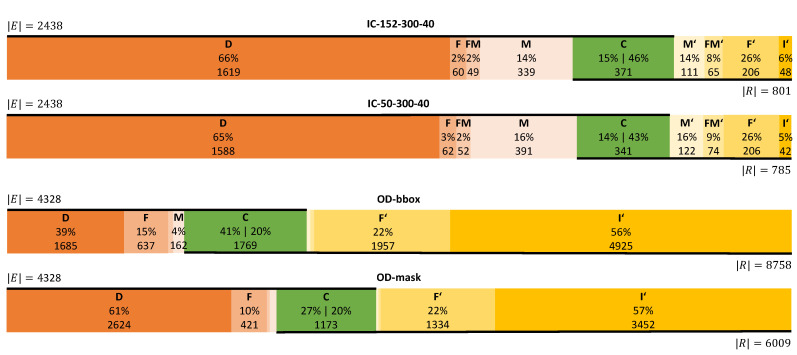
EAD diagrams visualizing the event based error classes with respect to ground truth events |E| and returned events |R|.

Further, we report event metrics which are normalized by the number of ground truth events |E| or the number of retrieved events |R| (see Figure 4). Both *IC* methods show a similar distribution of error classes. For IC-152-300-40, we observe a high fraction of deletions, $\frac{D}{\left|E\right|}=66.41\%$, and a low fraction of insertions, $\frac{I^{\prime }}{\left|R\right|}=5.99\%$, which is consistent to frame metrics. 371 predictions are correct which corresponds to Re=15.22% (conservative recall) of the ground truth and Pr=46.32% (conservative precision) of all retrieved events. The more progressive recall and precision is higher with $Re^{*}=33.59\%$ and $Pr^{*}=94.01\%$. The distribution of the remaining error classes shows, e.g., how many fragmenting events $F^{\prime }$ (206→25.72%) cause the fragmentations *F* (60→2.46%) in the ground truth.

The two *OD* methods have a similar distribution of error classes for retrieved events: the rate of fragmenting events is $\frac{F^{\prime }}{\left|R\right|}\approx 22\%$, the rate of insertions is $\frac{I^{\prime }}{\left|R\right|}\approx 56\%$, the counts for $M^{\prime }$ and $FM^{\prime }$ are very low, and the conservative precision Pr is similar with $\frac{C}{\left|R\right|}\approx 20\%$. However, OD-bbox predicts 2749 events more than OD-mask which results in a higher absolute number of correct events for OD-bbox (C=1769) compared to OD-mask (C=1173). With |E| being constant for both *OD* methods, $Re=\frac{C}{\left|E\right|}$ is higher for OD-bbox (40.88%) than for OD-mask (27.1%). Consequently, the fraction of deletions for OD-bbox (38.94%) is lower than the fraction for OD-mask (60.63%) which is close to the level of the *IC* methods. Further, the *OD* methods report a higher level of fragmented events *F* than merged events *M*. We observe the opposite for *IC*. The progressive precision and recall values are $Pr^{*}=43.77\%$ and $Re^{*}=61.06\%$ for OD-bbox, and $Pr^{*}=42.55\%$ and $Re^{*}=39.37\%$ for OD-mask.

#### 4.4.2. AOI Performance Breakdown

**Figure 5:**
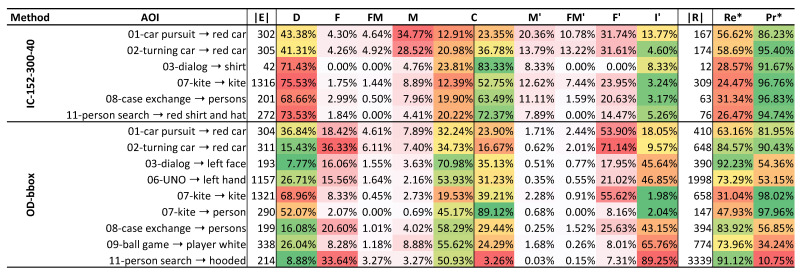
EAD table for AOIs with a non-zero recall ($dr<90\%;\frac{D}{\left|E\right|}\ll 100\%$) for IC-152-300-40 and OD-bbox.

For IC-152-300-40 and OD-bbox, we report the event metrics per AOI in a table (see Figure 5). For *IC*, we observe a difference between the two “red car” AOIs and the remaining AOIs. On average, we see a lower level of deletions for “red car” with $\frac{D}{\left|E\right|}=42.34\%$ and an increased level of merged events with $\frac{M}{\left|E\right|}=31.65\%$, compared to the other AOIs which average to 72.29% and 6.51%, respectively. Consequently, we observe the best progressive recall for “red car” with $Re^{*}=57.66\%$ on average, compared to $Re^{*}=27.71\%$ for the other AOIs. The conservative precision $\frac{C}{\left|R\right|}=30.07\%$ is lower than the average of 67.99% for the other AOIs. For “red car”, *M* is higher and *C* is lower for 01-car pursuit than for 02-turning car. Hence, the conservative recall $\frac{C}{\left|E\right|}=12.91\%$ for 01-car pursuit is relatively lower by 38.47%, while the $Re^{*}$ is lower by 3.53% only. Further, we observe the highest relative number of insertions with $\frac{I^{\prime }}{\left|R\right|}=13.77\%$ (others average to 4.92%). The *OD* methods result in more diverse error class distributions. We observe a low level of *D* in relation to ground truth events |E| for “red car” (02-turning car), “left face”, “persons”, and “hooded”, averaging to 12.04%. The AOIs “kite” and “person” result in the highest level for deletions *D* with 68.96% and 52.07%, respectively. For these AOIs, the low and high levels for *D* coincide with the highest and lowest $Re^{*}$ values. Overall, we see a high level of fragmented events *F* with highest values for “hooded” with 33.64% and “red car” in 02-turning car with 36.33%, and lowest values for “person” in 07-kite with 2.07%. The AOI “left face” results in the best conservative recall, Re=70.98%, due to the low level of deletions *D*, with 7.77%. For insertions $I^{\prime }$, we observe the lowest levels for the AOIs in kite with an average of 2.01%, followed by “red car” with 9.57% in 02-turning car and 18.05% in 01-car pursuit. All other AOIs average to 58.13% with a peak for “hooded” with 89.25%. These four AOIs with the lowest insertion rate $\frac{I^{\prime }}{\left|R\right|}$ have the best progressive precision with, on average, $Pr^{*}=92.09\%$. The remaining five AOIs average to $Pr^{*}=41.87\%$ with the minimum for “hooded” with $Pr^{*}=10.75\%$. The highest levels of fragmenting events are observed for “red car” (53.9% and 71.14%) and “kite” (55.62%). To compare the results of AOIs that remain for *IC* and *OD* methods, we calculate an event-based $f_{1}$ score as $f_{1}=2\cdot \frac{Pr^{*}\cdot Re^{*}}{Pr^{*}+Re^{*}}$. For the AOIs “red car” (x2), “kite”, and “persons” (08-case exchange), we receive $f_{1}$ scores of 68.36%,72.67%,39.06%,47.36% for IC-152-300-40 and 71.34%,87.4%,47.15%,67.78% for OD-bbox. For this selection of AOIs, OD-bbox yields the respectively better performance.

#### 4.4.3. Impact of IC Parameters on Performance

**Figure 6:**
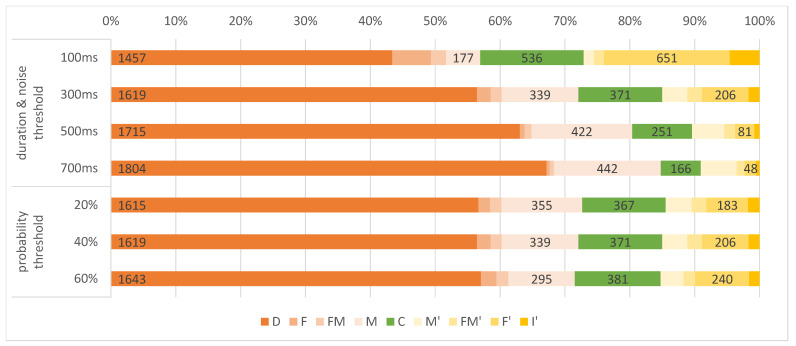
Simplified EAD diagrams for *IC* methods with varying parameters aggregated over AOIs with non-zero recall. We vary the probability threshold $T_{p}$ or the duration and noise threshold $T_{dur}=T_{noise}$. We use default values for other parameters. The results for 300 ms and 40% refer to the default setting IC-152-300-40 as shown in Figure 4.

The *IC* method offers multiple parameters for tuning the outcome, besides the classification *model*. We investigate the impact of $T_{dur}$ & $T_{noise}$ and $T_{p}$ on the frame and event metrics. With varying $T_{p}$, we observe no changes in the distribution of event error classes (see Figure 6). In addition, $Pr^{*}$ ranges between 32.69% and 33.76%, and $Re^{*}$ ranges between 93.58% and 94.39% for the different settings of $T_{p}$. When increasing the duration and noise thresholds, we observe a monotonic increase in the number of deletions *D*: the ratio $\frac{D}{\left|E\right|}$ ranges from 59.49% for 100 ms to 70.87% for 700 ms. At the same time, $\frac{M}{\left|E\right|}$ increases from 7.23% to 18.1% and $Re=\frac{C}{\left|E\right|}$ decreases from 21.89% to 6.8%. Concerning the error classes of retrieved events, we observe a monotonic decrease in the number of insertions $I^{\prime }$ ranging from 10.64% for 100 ms to 3.18% for 700 ms. Similarly, $\frac{F^{\prime }}{\left|R\right|}$ decreases from 44.99% to 11.74%, as well as the absolute number of retrieved events |R| which ranges from 1447 to 409. The level of merging events $M^{\prime }$ increases from 3.73% to 36.19% which corresponds to the increase of merged events *M*. In addition, we see a trend in the progressive precision and recall values: with increasing duration and noise threshold, $Re^{*}$ decreases from 40.51% to 26.13% and $Pr^{*}$ increases from 89.36% to 96.82%. The highest $f_{1}$ score of 55.74% is reached for 100 ms.

## 5. Discussion

Our results show that using our methods with default parameters and the AOI configuration from Table 1 does not support all AOIs. In particular, our observations confirm that they fail in detecting visual attention for AOIs without a mapping. This affects 15 AOIs (44.12%) for *IC* and 17 AOIs (50%) for *OD*. Our results reveal 13 additional AOIs for *IC* and 8 AOIs for *OD* with weak matches that result in zero or close to zero recalls (dr≥90%). Effectively, we count 28 AOIs (82.35%) for *IC* and 25 AOIs (73.53%) for *OD* as failing. We attribute these fails to challenge I, because the concepts of the AOIs have no adequate match to any class label of the underlying computer vision model. And, if there is a matching class label, the instances might differ from what the model has learned, i.e., from the training samples.

### 5.1. Overall Performance

**Figure 7:**
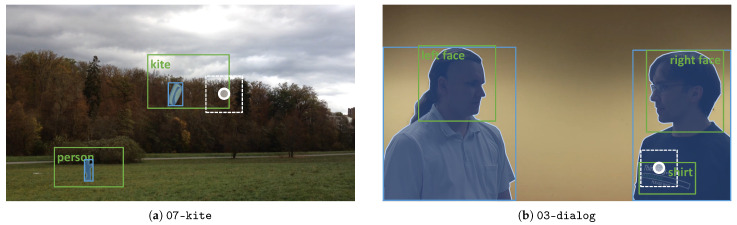
Example frames from two scenarios of the VISUS dataset [2] showing the recent fixation (white circle), ground truth annotations (green), object masks and bounding boxes for *OD* (blue), and the cropping area for *IC* (white rectangle).

The frame and event metrics for the remaining AOIs show that deletions are the most frequent false negative error across all methods. Overall, the frame-based deletion rates dr are lower than the respective level of deletion events *D*. For instance, in IC-152-300-40, $\frac{D}{\left|E\right|}=66.41\%$ of the ground truth events correspond to dr=36.79% of the positive ground truth frames. This may indicate that our methods delete more short events than long ones. The high level of deleted events might be caused by false negatives from the computer vision model which relates to challenge I. Another problem could be that our models fail in mapping the gaze signal although the prediction was correct. To investigate this issue further, we generated videos showing the manual annotations, the gaze and fixation events, and our prediction output. We noticed that the eye tracking signal frequently suffers from low accuracy and, hence, the gaze point does not hit an AOI object even though it is obvious that the participant followed that object, e.g., a “kite”. The manual annotations (bounding boxes) in the VISUS dataset are bloated up to include such erroneous gaze signals which better captures the human behavior than exact annotations. However, under the assumption that the gaze signal was accurate, this style of data annotation results in a lot of false positive ground truth events (see Figure 7a). Our methods are not robust against such cases, because they rely on local image classifications (*IC*) or fixation to object mask mapping (*OD*). Consequently, our methods report no attention events which might be one of the major reasons for the high level of deletions. We investigate this issue in detail in Section 5.4. This could also explain the difference between OD-bbox, using bounding boxes, and OD-mask, using exact object masks, i.e., OD-bbox better resamples the manual annotations and, to some degree, compensates the inaccurate gaze signals. Overall, OD-bbox shows the best progressive recall with $Re^{*}=61.06\%$, while the other methods average around 35.91%. Also, our results show that *OD* yields more insertion errors than *IC* in terms of frame and event metrics: the insertion rate is $\frac{I^{\prime }}{\left|R\right|}\approx 56\%$ for *OD* methods and 6% for *IC* methods. Consequently, with an average of $Pr^{*}=94.33\%$, *IC* results in the better progressive precision than *OD* with $Pr^{*}=43.16\%$. This suggests that the *IC* method may be the better choice for use cases with a good object to class label match, and if false negative errors are not severe. In addition, the relation of $FM,F,F^{\prime }$ to fr and $FM^{\prime },M,M^{\prime }$ to mr can reveal more about the error characteristics. For instance, if we see many event errors and a low ratio of corresponding frame errors, the fragmenting or merging predictions approximate the ground truth well (see Figure 2). For instance for IC-152-300-40, merge errors *M* make up 14% of the event errors with respect to the ground truth, but result in a low frame error rate of mr=0.89%.

### 5.2. Performance per AOI

The results for default parameters at the AOI level show that OD-bbox performs best for the four overlapping AOIs (see Figure 5). However, all other AOIs for OD-bbox suffer from high insertion levels of more than 40%. A reason might be that these AOIs match to “person” (see Table 1) and, at least, a second AOI shares this concept, which relates to challenge II. For instance, we map the MS COCO class label “person” to the AOI “left face” in 03-dialog, but “person” would also fit “right face” and “shirt”. The generated debug videos show that both *OD* methods detect attention events for “right face” and “shirt” based on the “person” class label. However, these are wrongly mapped to “left face” which results in a high number of false positives (see Figure 7b). This problem of the remaining AOIs is likely to cause the high level of insertion errors and the low progressive precision for *OD*, overall.

### 5.3. Impact of IC Parameters

Our investigation with different parameters for *IC* reveals that $T_{p}$ is likely to have no impact on event metrics. Our assumption is that the subsequent aggregation of image classification results is a harder criterion than a high $T_{p}$. E.g., an incorrect classification with low probability might be dropped anyway due to reaching $T_{noise}$, because it alternates with other wrong classifications. The parameters $T_{dur}$ and $T_{noise}$ have a clear impact on the performance: increasing the threshold results in decreasing values of Re and $Re^{*}$. $T_{dur}=T_{noise}=100$ ms yields the best overall performance by means of the $f_{1}$ score, followed by the default setting which results in a better $Pr^{*}$, but worse $Re^{*}$.

### 5.4. Impact of Re-Annotating the Ground Truth Data on Deletions

In many cases, the gaze recordings from the VISUS dataset suffer from a low spatial accuracy, which resulted in coarse manual annotations. For instance in Figure 7a, the manual annotations for “person” and “kite” (green bounding boxes) are much larger than the actual object to catch the point of gaze that, when looking at the video, obviously follows the kite. In contrast, the bounding boxes and exact object masks generated by Mask R-CNN (blue rectangles and polygons) frame the “person” and the “kite” closely. Our hypothesis is, that this kind of annotation is responsible for a large portion of deletion errors (false negative events), because the ground truth reports a false attention event that cannot be captured by our detection methods. To verify our assumption, we re-annotate AOIs without a close to zero recall (see Figure 5) and repeat our analysis using the new ground truth annotations, but the same event predictions from IC-152-300-40 and OD-bbox that we have gathered in our main experiment. The videos are annotated by a single annotator and reviewed by an eye tracking expert using the Computer Vision Annotation Tool CVAT (https://github.com/openvinotoolkit/cvat, accessed on 15 June 2021). We use the polygon-based annotation feature: a polygon is created that closely frames an object at keyframes with interpolation for intermediate frames. The results show that the ratio of deletion events $\frac{D}{\left|E\right|}$ decreases by 16.3% to 50.11% for the *IC* method and by 10.3% to 28.64% for the *OD* method. Consequently, the progressive recall values $Re^{*}$ increase by the same amounts to 49.89% for *IC* and to 71.36% for *OD*. Thus, we can confirm our hypothesis that coarse AOI annotations increase the level of deletions. This emphasizes the importance of accurate gaze estimation methods to avoid such errors. Further, it raises the need for error-aware gaze-to-object mapping methods to compensate the impact of the gaze estimation error, similar to those presented in Barz et al. [5]. For instance, we could detect an AOI hit by checking whether the distance of a fixation point to the boundary of an AOI is smaller than a defined threshold.

### 5.5. Limitations & Future Work

Our evaluation revealed several limitations that relate to the challenges that we identified in Section 4.1.1 or to accuracy issues with the gaze signal in the VISUS dataset. The main limitation of our methods is related to challenge I: many AOIs are not supported because the concepts are not included with the pre-trained computer vision models. A promising solution to address it is to collect new samples for unsupported AOIs and AOIs with weak matches for fine-tuning the computer vision models [75,76]. We want to investigate the effectiveness of interactive machine learning methods for this purpose [77,78] compared to randomly annotating a small portion of the data as suggested in Wolf et al. [16]. Training a model from scratch, as suggested in [11,12], is not an option with state-of-the-art computer vision models, because they need a large quantity of training samples. Further, our methods offer no solution for challenge II: AOIs share the same concept. This could be solved using similarity models with interactive training. For instance, we could iteratively train a model to differentiate between “left face” and “right face” which would reduce the number of insertion errors for 03-dialog. Using multiple object tracking algorithms [57] with humans-in-the-loop is a promising approach to support challenges III and IV. Further, we plan to develop error-aware gaze to AOI mapping similar to [5,79] to compensate for the gaze estimation error in mobile eye tracking. Also, it is likely that the fixation detection algorithm used in [2] has an impact on the ground truth extraction. The authors mentioned that, e.g., smooth pursuit movements are not supported well, which make up a large portion of the data. The selection of a suitable fixation detection algorithm is even more important for mobile eye tracking [74]. In addition, we recently showcased a real-time application of the *IC* method in an augmented reality setting with objects that are well represented in the training data of the image classification model [72], similar to Machado et al. [9], based on the AR eye tracking toolkit [80]. Our method enables a stable augmentation of ambient objects via the head-mounted display.

## 6. Conclusions

In this work, we implemented two methods for detecting visual attention using pre-trained deep learning models from computer vision. In addition, we defined an evaluation framework based on the VISUS dataset by Kurzhals et al. [2] and identified four challenges for methods that map gaze to AOIs. We used a set of fine-grained metrics by Ward et al. [18] from the field of activity recognition to evaluate our visual attention to AOI mapping methods. Our methods performed well for AOIs with distinct concepts which have a strong match to the pre-trained model classes. However, several limitations impede our goal of accelerating and objectifying AOI annotation in eye tracking research. For instance, our methods drop in performance when a concept is not supported, when two instances of the same concept cannot be disambiguated, or when gaze estimation errors occur. In the discussion, we proposed ways to overcome these limitations. In particular, we suggest to use interactive machine learning for adapting our methods to new scenarios or to differentiate between instances of the same concept. Further, we proposed an approach based on multi-object tracking to cope with AOIs that have a similar appearance.

## Figures and Tables

**Table 1:** Overview of scenarios and AOIs in the VISUS dataset and the corresponding mappings of class labels to AOIs. Class labels originate from ImageNet in case of *IC* methods and from MS COCO in case of *OD* methods.

Scenario	AOI	ImageNet Labels	MS COCO Labels
01-car pursuit (25 s)	red car	streetcar, sports car, minivan, cab, minibus, limousine, car mirror, racer, passenger car	car
	white car	–	–
02-turning car (28 s)	red car	streetcar, sports car, minivan, cab, minibus, limousine, car mirror, racer, passenger car	car
03-dialog (19 s)	left face	ear	person
	right face	–	–
	shirt	sweatshirt	–
04-thimblerig (30 s)	cup1	cocktail shaker, coffee mug, cup	cup
	cup2	–	bowl
	cup3	–	–
05-memory (148 s)	cards	desk	dining table
06-UNO (121 s)	left hand	–	person
	right hand	–	–
	stack covered	desk	dining table
	stack uncovered	–	–
07-kite (97 s)	person	lab coat, poncho, cardigan, cloak, sweatshirt, trench coat	person
	kite	balloon, kite, parachute	kite
08-case exchange (27 s)	persons	sombrero, cowboy hat	person
	textbox	–	–
	case	mailbag, packet, plastic bag, shopping basket, backpack, bucket, crate	handbag, suitcase
	suspects	lab coat, poncho, cardigan, cloak, sweatshirt, trench coat	–
09-ball game (31 s)	ball	baseball, basketball, rugby ball, tennis ball, volleyball, soccer ball	sports ball
	player white	ballplayer	person
	player red1	–	–
	player red2	–	–
	player red3	–	–
10-bag search (133 s)	red bag	plastic bag	handbag
	yellow bag	–	–
	blue bag	–	–
	red-white bag	–	–
	brown bag	mailbag	–
	persons	lab coat, poncho, cardigan, cloak, sweatshirt, trench coat	person
11-person search (172 s)	hooded	lab coat, poncho, cardigan, cloak, sweatshirt, trench coat	person
	red shirt and hat	sombrero, cowboy hat	–
	persons	–	–

## Data Availability

The VISUS dataset is provided online by Kurzhals et al. [2] at https://www.visus.uni-stuttgart.de/publikationen/benchmark-eyetracking (accessed on 12 April 2021). In addition, we provide the extracted ground truth events and predicted events for each scenario and participant as supplementary material.

## Abbreviations

**General**	
AOI	Area of Interest
IC	[Method] Detect Attention using Gaze-guided Image Classification
OD	[Method] Detect Attention using Object Detection
EAD	Event Analysis Diagram

**Event-based Error Classes and Metrics**	
|E|	Number of ground truth events |E|=D+F+FM+M+C
|R|	Number of predicted events, or returns $\left|R\right|=M^{\prime }+FM^{\prime }+F^{\prime }+I^{\prime }+C$
*D*	Number of deletion errors (false negatives)
$I^{\prime }$	Number of insertion errors (false positives)
$F,F^{\prime }$	Number of fragmentation errors
$M,M^{\prime }$	Number of merge errors
$FM,FM^{\prime }$	Number of fragmentation and merge errors (if both occur together)
*C*	Number of correct events
Pr	Event-based precision $Pr=\frac{C}{\left|R\right|}$ (conservative)
Re	Event-based recall $Re=\frac{C}{\left|E\right|}$ (conservative)
$Pr^{*}$	Event-based precision $Pr^{*}=\frac{\left|R\right|-I^{\prime }}{\left|R\right|}$ (progressive)
$Re^{*}$	Event-based recall $Re^{*}=\frac{|E|-D}{\left|E\right|}$ (progressive)

**Frame-based Error Classes and Metrics**	
*P*	Total number of positive frames
*N*	Total number of negative frames
TP	Number of true positives (frames)
TN	Number of true negatives (frames)
$D_{f},dr$	Number of deletion errors (frames), deletion rate
$I_{f},ir$	Number of insertion errors (frames), insertion rate
$F_{F},fr$	Number of fragmentation errors (frames), fragmentation rate
$M_{f},mr$	Number of merge errors (frames), merging rate
$O_{f},o$	Number of overfill errors (frames), ratio of overfills
$U_{f},u$	Number of underfill errors (frames), ratio of underfills
tpr	True positive rate
fpr	False positive rate
